# Fabrication and Cell Culture Applications of Core‐Shell Hydrogel Fibers Composed of Chitosan/DNA Interfacial Polyelectrolyte Complexation and Calcium Alginate: Straight and Beaded Core Variations

**DOI:** 10.1002/adhm.202302011

**Published:** 2023-08-03

**Authors:** Yoshinobu Utagawa, Kosuke Ino, Masahiro Takinoue, Hitoshi Shiku

**Affiliations:** ^1^ Graduate School of Engineering Tohoku University Sendai 980–8579 Japan; ^2^ Department of Computer Science Tokyo Institute of Technology Yokohama 226–8502 Japan; ^3^ Graduate School of Environmental Studies Tohoku University Sendai 980–8579 Japan

**Keywords:** cell cultures, chitosan complex, core‐shell hydrogel fibers, DNA complex, interfacial polyelectrolyte complexation fibers

## Abstract

Core‐shell hydrogel fibers are widely used in cell culture applications. A simple and rapid method is presented for fabricating core‐shell hydrogel fibers, consisting of straight or beaded core fibers, for cell culture applications. The core fibers are prepared using interfacial polyelectrolyte complexation (IPC) with chitosan and DNA. Briefly, two droplets of chitosan and DNA are brought in contact to form an IPC film, which is dragged to prepare an IPC fiber. The incubation time and DNA concentration are adjusted to prepare straight and beaded IPC fibers. The fibers with Ca^2+^ are immersed in an alginate solution to form calcium alginate shell hydrogels around the core IPC fibers. To the best of the knowledge, this is the first report of core‐shell hydrogel fibers with IPC fiber cores. To demonstrate cell culture, straight hydrogel fibers are applied to fabricate hepatic models consisting of HepG2 and 3T3 fibroblasts, and vascular models consisting of human umbilical vein endothelial cells and 3T3 fibroblasts. To evaluate the effect of co‐culture, albumin secretion, and angiogenesis are evaluated. Beaded hydrogel fibers are used to fabricate many size‐controlled spheroids for fiber and cloning applications. This method can be widely applied in tissue engineering and cell analysis.

## Graphical abstract

Fabrication and cell culture applications of core‐shell hydrogel fibers composed of chitosan/DNA interfacial polyelectrolyte complexation and calcium alginate are presented. Beaded fibers and straight fibers are successfully fabricated using different DNA concentrations. In addition, it is demonstrated that these hydrogel fibers can be used for cell culture applications.

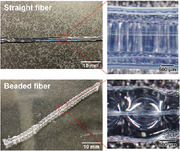

## 1 Introduction

Hydrogels are widely used for 3D cell culture because of their useful properties such as high water content, high permeability, and biocompatibility. For example, calcium alginate (Ca‐alginate) hydrogels have been extensively used for cell culture due to their simple fabrication process via a reaction between alginate and Ca^2+^.^[^ ^1^ ^]^ Hydrogels with various structures, such as fibers, beads, and sheets, have been developed for specific organs and tissues. In particular, hydrogel fibers have attracted attention in cell culture because the human body comprises several types of fiber structures, such as blood vessels and muscles. In tissue‐engineering applications, hydrogel fibers are created using techniques such as microfluidics, 3D bioprinting,^[^ ^2^ ^]^ and electrochemical techniques.^[^ ^3^ ^]^ Microfluidic devices are commonly used to prepare complex fibers. For example, a microfluidic device with multiple inlet channels was used to fabricate core‐shell fibers of extracellular fibers with cells and Ca‐alginate.^[^ ^4^ ^]^ In the previous study, islet cell fibers in the core were encapsulated in a mechanically stable alginate hydrogel as the shell, and the fibers were transplanted into diabetic mice. The same group reported that beta cells were cultured for diabetes treatment and that the alginate hydrogel shell protected beta cells from immune cells.^[^ ^5^ ^]^ Cancer invasion model have been reported using core‐shell hydrogel fibers with micropassage.^[^ ^6^ ^]^ Human induced pluripotent stem cells have also been cultured in alginate core‐shell hydrogels, resulting in sustained high viability and pluripotency.^[^ ^7^ ^]^ Thus, microfluidic systems are very useful for the fabrication of core‐shell hydrogel fibers, but they require specific equipment for preparation and control. Then, other methods were reported not to require microfluidic devices. Alginate shells were fabricated using sacrificial templates of sugar, which can culture cells in the cores.^[^ ^8^ ^]^ In addition, alginate shells could be also fabricated using an electrochemical reaction to generate Ca^2+^.^[^ ^9^ ^]^ This system can control the shell thickness by the time of the electrochemical reaction. However, these methods require to introduce cells into the cores finally. Therefore, a simpler approach is required.

An interfacial polyelectrolyte complexation (IPC) technique was proposed for the simple and rapid fabrication of hydrogel fibers.^[^ ^10^ ^]^ The IPC film was fabricated at the interface of polymer solutions with opposite charges, and the IPC fiber was prepared by dragging the film upward. IPC fibers have been employed in 3D cell culture because they are fabricated under mild conditions, such as room temperature, neutral pH, and aqueous solution. These fibers easily incorporate extracellular matrix proteins and modify peptides.^[^ ^11^ ^]^ Previously, chitosan/alginate^[^ ^12^ ^]^ and chitin/alginate^[^ ^13^ ^]^ IPC fibers were used for cell culture. In addition, a multiple‐fiber combination model has been reported.^[^ ^14^ ^]^ This model can be used to fabricate co‐culture models with the alignment of multiple cell types. Moreover, chitosan/heparin IPC fibers that can be utilized as biocompatible functional sutures have been reported.^[^ ^15^ ^]^ Apart from alginate, DNA, an anionic polymer, has been used in IPC fibers.^[^ ^16^ ^]^ DNA can be modified in various ways, which are expected to improve the function of IPC fibers. Thus, IPC fibers can be fabricated using various combinations of polymer solutions. In addition, beaded fibers can be easily fabricated by adjusting the experimental conditions because the fibers are prepared in air, and droplets form spontaneously on the fibers, although it is difficult to fabricate beaded fibers using microfluidic devices. Therefore, IPC fibers have excellent features; however, to the best of our knowledge, core‐shell hydrogels using IPC fibers have not been reported.

In the present study, we present a simple and rapid process using IPC fibers to fabricate chitosan/DNA core fiber‐embedded Ca‐alginate shell hydrogels for cell culture. This method requires only three types of polymer solutions and can be carried out at room temperature, without the need for complex and expensive equipment. Furthermore, hydrogel fibers with straight cores were used for the creation of co‐culture models for cell culture. A hepatic model was constructed by co‐culturing hepatoblastoma cells and fibroblasts, and albumin production was evaluated in monoculture and co‐culture models. Additionally, a vascular model was established by co‐culturing vascular endothelial cells and fibroblasts, and angiogenesis was evaluated in both the monoculture and co‐culture models. Finally, beaded IPC cores embedded in Ca‐alginate shell hydrogels were used for size‐controlled spheroidal culture and cell‐cloning applications. This is the first report of core‐shell structures using IPC fibers for cell culture applications. In particular, the use of beaded IPC fibers for cell culture was reported for the first time. Overall, this study presents a novel and efficient method for fabricating core‐shell hydrogel fibers for cell culture, which has significant potential for use in tissue engineering and regenerative medicine.

## 2 Results and Discussion

### 2.1 Fabrication Scheme

**Figure 1:**
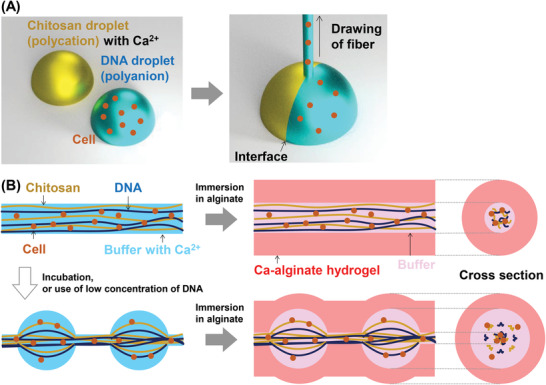
Schematic of the fabrication process of core‐shell hydrogel fibers of chitosan/DNA IPC and Ca‐alginate for cell culture applications. A) Fabrication process of chitosan/DNA IPC fibers. B) Fabrication process of core‐shell hydrogel fibers. The top illustration shows the fabrication of straight core fibers, while the bottom illustration shows the fabrication of beaded core fibers. By incubating or using a low DNA concentration, a droplet array forms at the fiber.

**Figure** **1** depicts the fabrication process of core‐shell hydrogel fiber‐containing cells. DNA, a commercially available and biocompatible material, was selected as the anionic polymer due to its high programmability and multifunctionality, which makes it widely used in biomedical and bioanalysis applications.^[^ ^17^ ^]^ Capsule‐like DNA hydrogels were formed by the lateral phase separation of DNA nanostructures.^[^ ^18^ ^]^ In this study, we developed an IPC fiber using a chitosan droplet containing Ca^2+^ and a DNA droplet containing cells (Figure 1A). After fabrication, the straight IPC fibers were immediately immersed in an alginate solution (Figure 1B) to form a Ca‐alginate shell hydrogel outside the core fibers.

In contrast, when a low concentration of DNA was used, the low‐viscosity solution in the IPC fiber spontaneously formed beads due to its surface tension, resulting in the successful formation of beaded IPC fibers (Figure 1B). The beaded fibers containing cells and Ca^2+^ were then immersed in an alginate solution to prepare a Ca‐alginate shell hydrogel. The experimental section provides a detailed description of the experimental conditions.

### 2.2 Chitosan/DNA IPC Fibers

**Figure 2:**
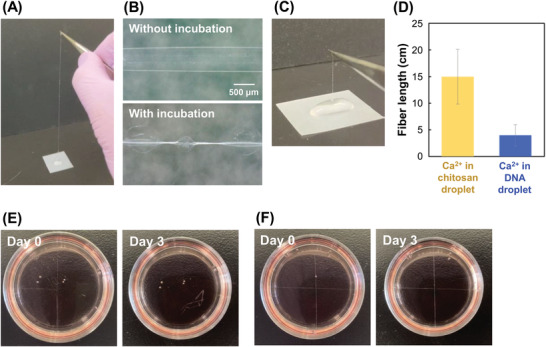
Chitosan/DNA IPC fibers. A) Photograph of the fiber during fabrication. Time‐lapse images are shown in Movie S1 (Supporting Information). B) Microscopic image of the fibers without or with incubation. DNA: 1.0% (w/v) without Ca^2+^ in (A) and (B). The brightness of the images was adjusted. C) Photograph of the fiber when using the DNA droplet with Ca^2+^. D) Fiber length when Ca^2+^ was in a chitosan or DNA droplet. The error bars represent the standard deviations. *n* = 9. Time‐lapse images of E) the fibers and F) chitosan/alginate IPC fibers as a control.

As shown in **Figure** **2**A and Movie S1 (Supporting Information), IPC fibers without Ca^2+^ were very rapidly fabricated without complex equipment, compared to other approaches such as the microfluidic approach. The diameter was <1 mm (Figure 2B) with a large range because the fibers were fabricated manually. Then, the beads were formed when incubated in air for 60 s. The length was over 1 m using the 100 µL droplets within 1 min, and the length could be improved by modifying the volume of the droplets. The influence of the distance of droplets on fiber formation is shown in Figure S1 (Supporting Information) and discussed in the Supporting Information.

Next, we investigated the effect of Ca^2+^ on IPC fiber formation. The IPC fiber was successfully fabricated from a DNA droplet containing Ca^2+^ (Figure 2C; Movie S2, Supporting Information), but the fiber length was significantly reduced from over 100 to 4 cm. This was because the electrostatic interaction between Ca^2+^ and DNA inhibited the interaction between chitosan and DNA. In contrast, when chitosan droplets with Ca^2+^ were used, the fiber length increased to 15 cm (Figure 2D). Thus, Ca^2+^ was added to the chitosan droplets to fabricate Ca‐alginate shell hydrogels.

We also investigated the stability of the chitosan/DNA IPC fibers. After incubation in culture medium at 37 °C for 3 days, the chitosan/DNA fibers were dissolved (Figure 2E). In contrast, the structure of chitosan/alginate fibers was maintained (Figure 2F). These results indicate that DNA is more easily decomposed than alginate, which is widely used for IPC. This is a merit because DNA fibers can be replaced with cells during culture. However, chitosan/DNA IPC fibers without shells are unsuitable for long‐term culture. Therefore, Ca‐alginate shell hydrogels were prepared around chitosan/DNA IPC fibers for long‐term culture.

### 2.3 Core‐Shell Hydrogel Fiber of Chitosan/DNA and Ca‐Alginate

**Figure 3:**
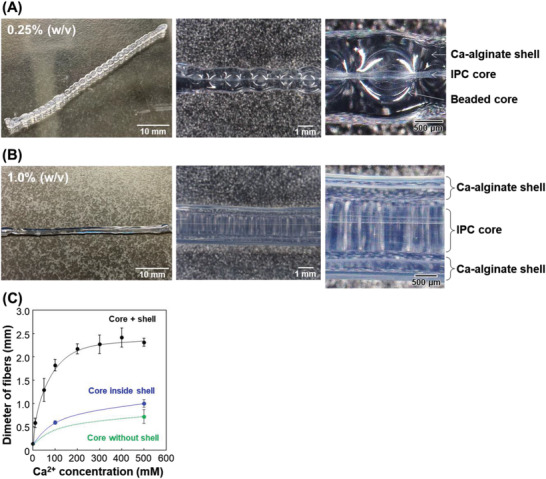
Core‐shell hydrogel fiber of Chitosan/DNA IPC and Ca‐alginate when using A) 0.25 and B) 1.0% (w/v) DNA. Ca^2+^: 500 mm. Left: whole images. Middle: microscopic images. Right: magnified images of the middle. C) Diameter of the hydrogel fibers with various Ca^2+^ concentrations. DNA: 1.0% (w/v). Blue and green symbols represent the core diameter inside the shell and the diameter of the core fabricated without the shell, respectively. The blue and green lines are based on predicted diameter lengths. The error bars represent the standard deviations. *n* = 3.

As shown in **Figure** **3**, core‐shell hydrogel fibers of chitosan/DNA and Ca‐alginate were successfully created in ≈5 min. This indicates that there was sufficient diffusion of Ca^2+^ from the fibers to the alginate solution, leading to the rapid formation of Ca‐alginate hydrogels near the interface of the alginate solution and the IPC fibers. The diameter of the core‐shell hydrogel fibers was ≈2 mm. Interestingly, the DNA concentration had a considerable impact on the shape of the fibers, as the solution viscosity was closely related to the formation of beads on IPC fibers.^[^ ^10^ ^]^ With a low‐viscosity solution of 0.25% (w/v) DNA, a beaded IPC fiber was created as a core‐embedded Ca‐alginate shell hydrogel (Figure 3A). The hydrogel fiber consisted of a chitosan/DNA IPC core, a beaded core, and a Ca‐alginate shell, as illustrated in Figure 1B. The large beads were spaced out in roughly equal intervals (5.58 ± 0.48 beads cm^−1^), with a diameter of 0.92 ± 0.09 mm. Small beads were often present between the large beads. In contrast, using a high‐viscosity solution of 1.0% (w/v) resulted in straight IPC fibers. After a 60‐s incubation period, the fiber shape changed from straight to beaded due to surface tension. When these straight IPC fibers were immediately immersed in the alginate solution, straight core‐shell hydrogel fibers were successfully produced (Figure 3B). Due to the slow diffusion rates of the polymers and nuclear fibers, bead formation required time, which allowed us to harden the IPC fibers with alginate hydrogels before bead formation. After alginate hydrogel formation, the nuclear fibers did not gather because the solution could not move during bead formation. However, when using a low‐concentration DNA solution, beads formed rapidly due to the rapid diffusion of polymers and nuclear fibers.

Next, the effect of Ca^2+^ concentration on the diameter of the core‐shell fibers was investigated. We found that the diameter increased with increasing Ca^2+^ concentrations and remained almost constant at ≈2.3 mm (Figure 3C). As the Ca^2+^ concentration increased, the core diameter inside the shell also increased (Figure 3C); however, the detailed mechanism is unclear. This increase in diameter may be caused by the weakening of the interaction between DNA and chitosan because of increased Ca^2+^ concentration. Compared to the diameter of the core fabricated without the shell, the diameter of the core inside the shell was large, indicating that shell formation and/or the increase in water amount around the core affected its diameter. At 500 mm Ca^2+^, the thickness of the shell was ≈660 µm, which was larger than that at 10 mm (100 µm) because the large amount of Ca^2+^ diffused to a Na‐alginate solution from the core, thereby forming a thicker Ca‐alginate hydrogel shell. Our results showed that Ca^2+^ concentration inside the core can impact shell thickness. We selected 500 mm for subsequent experiments due to the good stability of the fibers.

### 2.4 Cell Culture Applications Using Straight Fibers

**Figure 4:**
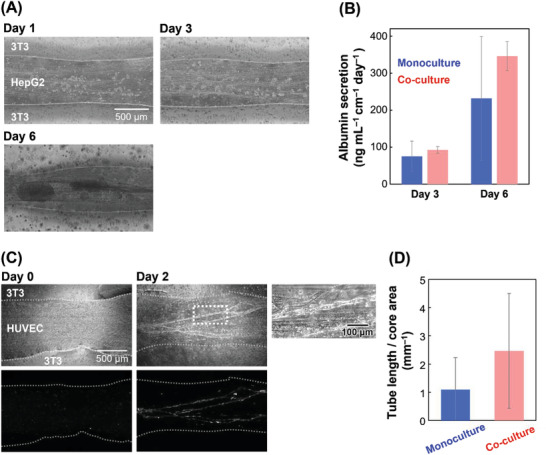
Cell culture applications using the straight fibers surrounding the Ca‐alginate shell. A,B) Hepatic model consisting of HepG2 in the core and 3T3 fibroblasts in the shell. A) Phase‐contrast images at days 1, 3, and 6. B) Albumin secretion from the fibers at days 3 and 6. The error bars represent the standard deviations. *n* = 4. C,D) Vascular model of RFP‐HUVEC in the core and 3T3 fibroblasts in the shell. C) Phase‐contract (top) and fluorescent images (bottom) at days 0 and 2. D) Tube length/core area. The error bars represent the standard deviations. *n* = 6–7.

In in vitro hepatocyte culture, the cellular functions of 3D culture are better than that of 2D culture.^[^ ^19^ ^]^ Furthermore, this functionality improves when co‐cultured with other cell types. Thus, 3D co‐culture models of fibroblasts^[^ ^20^ ^]^ or vascular endothelial cells^[^ ^14b^ ^]^ have been reported. Additionally, hepatic cells and fibroblasts have been incorporated in core‐shell hydrogel fibers.^[^ ^21^ ^]^ In the present study, a hepatic model consisting of HepG2 and 3T3 cells in the core and shell of the fibers, respectively, was fabricated, and its functions were evaluated. The effect of Ca^2+^ and pH on cell viability is discussed in Figure S2 (Supporting Information). The effect of cell concentration in droplets on the fiber length was discussed in Figure S3 (Supporting Information). The cell concentration for the shell is also discussed in the Supporting Information. **Figure** **4**A displays images of co‐cultured HepG2 and 3T3 cells. HepG2 and 3T3 cells were observed in the chitosan/DNA IPC core fibers and Ca‐alginate shell hydrogels, respectively. Although the HepG2 cells were uniformly dispersed in the core on day 1, cell clusters formed around day 6 (Figure 4A), indicating successful culture of HepG2 cells. In contrast, there was no direct physical interaction between the HepG2 and 3T3 cells at the core–shell interface. The model was created within 5 min without complex equipment, indicating that its speed and simplicity are superior to conventional methods for core‐shell hydrogels with cells, such as 3D bioprinting.^[^ ^22^ ^]^ Next, albumin secretion was measured from both monoculture and co‐culture fibers because albumin production is an indicator of liver function.^[^ ^23^ ^]^ Generally, the co‐culture of 3T3 cells has been reported to increase albumin production in HepG2 cells.^[^ ^24^ ^]^ Figure 4B shows that albumin concentrations increased over time in both the monoculture and co‐culture samples, indicating that the fibers might be effective in culturing cells and creating a hepatic model. The co‐culture might tend to increase albumin production on day 6, although the difference was not statistically significant (*p*‐value = 0.23; Student's *t*‐test). This is because HepG2 cells are stimulated by liquid factors produced by 3T3 cells. Thus, the fibers were used to fabricate functional double‐layer hydrogel fibers.

Vascularization is crucial in tissue engineering for maintaining tissue organization.^[^ ^25^ ^]^ To evaluate angiogenesis, human umbilical vein endothelial cells (HUVECs) and 3T3 cells were co‐cultured with a chitosan/DNA IPC core and a Ca‐alginate shell in the hydrogel fiber. Collagen was incorporated into the core fibers to effectively induce angiogenesis. After two days of culture, many vascular tubes were formed in the IPC fibers (Figure 4C). HUVECs were known to form tube structures in collagen matrix by self‐assembling.^[^ ^26^ ^]^ In addition, many tubes were aligned in the fiber direction because the cells are spatially controlled by nuclear fibers. The length of the vascular tubes was measured in HUVEC monocultures and HUVEC and 3T3 cell co‐cultures using ImageJ software. The results showed that the co‐culture might tend to have more angiogenesis than the monoculture (Figure 4D), although there is no significant difference (*p*‐value = 0.17; Student's *t*‐test), indicating that fibroblast growth factor (FGF) released from 3T3 cells might promote angiogenesis. This result suggests that the multiple vascular structures in the hydrogel fibers enable the long‐term culture of larger tissues. To prepare a simple vascular tube, narrow hydrogel fibers must be prepared by optimizing the experimental conditions for fiber fabrication.

### 2.5 Cellular Spheroids in an Array in a Single Fiber

**Figure 5:**
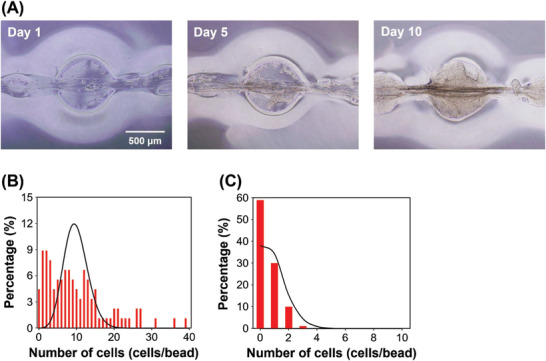
Cell culture applications using the beaded cores surrounded by the Ca‐alginate shell. A) Micrographic images of MCF‐7 cells cultured for 10 days in the beaded core. DNA: 0.25% (w/v). B,C) Histogram of cell numbers of MCF‐7 cells in the bead cores. The initial cell seeding densities were B) 1.0 × 10^5^ and C) 1.0 × 10^4^ cells mL^−1^. The average values were B) 9.9 and C) 0.9 cells bead^−1^. The lines show the Poisson distribution.

MCF‐7 cells were cultured in the core of the fibers to demonstrate the application of beaded fibers in cell culture. The cells successfully proliferated inside the beads, forming single cellular spheroids within each bead (**Figure** **5**A). The cells were unable to digest the Ca‐alginate shell, resulting in spheroids that were the same size as the beads. These results indicate that beaded fibers can form many spheroids with diameters similar to those of the beads.

The beaded fibers were then used to trap single cells into individual beads, which were used for single‐cell analyses such as cloning. Cloning involves the detection and identification of cells with specific DNA sequences in a generally engineered cell population to produce a cell population of the same genotype. Beaded fibers were fabricated using two different cell concentrations, and the number of cells in the beads was counted. Figure 5B shows a histogram of an average cell count of 9.9 cells bead^−1^. However, the cell numbers did not follow a Poisson distribution due to uneven bead sizes. In particular, there were many beads with 0–3 cells due to the formation of small beads between large beads. Figure 5C shows a histogram of the average cell count of 0.9 cells bead^−1^, with many beads without cells. The cell count was less than one cell in ≈90% of the beads, indicating that the beaded fibers could be used for cloning. Compared to the conventional use of microwells in cloning,^[^ ^27^ ^]^ hydrogel fibers do not require special equipment. In addition, they are easily handled because single cells can be encapsulated in beads and hardened using Ca‐alginate hydrogels. The beads can be completely separated from each other by cutting for preventing cross‐contamination.

## 3 Conclusion

In this study, we proposed a novel method for the fabrication of core‐shell hydrogel fibers for cell culture applications, based on IPC formation. Our fibers consisted of a chitosan/DNA core and a Ca‐alginate shell. First, we produced chitosan/DNA fiber‐embedded Ca‐alginate hydrogels. By adjusting the DNA concentration, we controlled the shape of the fibers and successfully created both straight and beaded fibers. To demonstrate the use of straight core‐shell hydrogel fibers in cell culture applications, we co‐cultured HepG2 and 3T3 cells in the straight hydrogel fibers and created a hepatic model. Our findings suggest that albumin production increased in the co‐culture compared to that in the monoculture. Additionally, a vascular model was established by co‐culturing HUVECs and 3T3 cells, and we found that angiogenesis may have been promoted in the co‐culture compared to that in the monoculture. To demonstrate the use of beaded fibers, spheroids were produced in an array of single fibers. DNA exhibits great potential as a scaffold for cell culture, drug delivery, and sensor development, owing to its modifiability. Our proposed methodology has broad utility over a wide range of bio‐applications.

## 4 Experimental Section

### Fabrication of Chitosan/DNA IPC Fibers

A 0.5% (w/v) chitosan solution (Sigma‐Aldrich) was dissolved in acetate solution (pH 5.6). DNA (0.5% [w/v]) from salmon testes (Sigma‐Aldrich) or 0.5% (w/v) sodium alginate (FUJIFILM Wako Pure Chemicals Corporation, Japan) was dissolved in Milli‐Q water. Next, 100 µL droplets of these solutions were prepared on a hydrophobic film, and two droplets were contacted to form an IPC film. The IPC fibers were formed by manually pulling the film up using a pair of tweezers at a speed of ≈1–5 cm s^−1^.

### Fabrication of Core‐Shell Hydrogel Fibers of Chitosan/DNA IPC and Ca‐Alginate Hydrogels

IPC fibers were produced using a 0.5% (w/v) chitosan solution containing 500 mm CaCl_2_ (FUJIFILM Wako Pure Chemicals Corporation) and 0.25 or 1.0% (w/v) DNA solution. Beaded and straight fibers were formed using 0.25 or 1.0% (w/v) DNA solutions, respectively. The IPC fibers were then immersed in a 1.5% (w/v) alginate solution in Dulbecco's phosphate‐buffered saline (PBS; Nacalai Tesque Inc., Japan) for 5 min.

### Cell Culture

HepG2 (human liver cancer cell line, American Type Culture Collection (ATCC), USA) and 3T3 (mouse fibroblast cell line, ATCC) cells were cultured in Dulbecco's modified Eagle's medium (Gibco, USA) supplemented with 10% fetal bovine serum (FBS, Gibco) and 1% penicillin‐streptomycin (PS, Gibco). Red fluorescent protein‐expressing human umbilical vein endothelial cells (RFP‐HUVECs; Angio‐Proteomie, USA) were cultured in endothelial growth medium (endothelial cell growth medium 2; Promo Cell, Germany) containing 1% PS. MCF‐7 cells (Institute of Development, Aging, and Cancer, Tohoku University, Japan) were cultured in RPMI1640 (Gibco) supplemented with 10% FBS and 1% PS. Cells were maintained at 37 °C in a humidified atmosphere containing 5% CO_2_. Phase‐contrast and fluorescence images were captured using a microscope (Olympus IX71; Nikon ECLOPSE Ti2; Japan).

### Core‐Shell Hydrogel Straight Fibers for Hepatic and Vascular Models

In the hepatic model, a cell suspension of 5.0 × 10^6^ cells mL^−1^ of HepG2 cells was used with a 1.0% (w/v) DNA solution. In the vascular model, a cell suspension of 5.0 × 10^6^ cells mL^−1^ of HUVECs was mixed with 1 mg mL^−1^ collagen with the DNA solution. After IPC fiber formation, the straight fibers in the alginate solution containing 5.0 × 10^5^ cells mL^−^^1^ of 3T3 cells were immersed for 5 min in a 5% CO_2_ atmosphere at 37 °C. The hydrogel fibers were then washed with PBS and cultured.

### Albumin Measurement Using ELISA

HepG2 and 3T3 cells were cultured in the fibers for three days, after which the medium was replaced with serum‐free medium. The medium was collected after 24 h. This process was repeated for six days. The albumin levels in the collected medium were measured using a human albumin ELISA kit (Bethyl Laboratories, Inc., USA). The absorbance of the samples was measured using a microplate reader (SpectraMax iD5, Molecular Devices, USA).

### Evaluation of Vascular Formation Using ImageJ

The evaluation of angiogenesis was performed using ImageJ software. HUVECs were cultured for 2 days in straight fibers, and phase‐contrast and fluorescence images were captured. The areas of the IPC fibers were calculated using phase‐contrast images. The total lengths of the vascular tubes were measured using fluorescence imaging.

### Core‐Shell Hydrogel Beaded Fibers for Culture of Spheroid Arrays and Single Cells

A cell suspension (5.0 × 10^6^ cells mL^−1^) of MCF‐7 cells in a 0.25% (w/v) DNA solution was used. After the formation of IPC fibers using a 0.5% (w/v) chitosan solution with 500 mm CaCl_2_, the beaded fibers were immersed in an alginate solution for 5 min in a 5% CO_2_ atmosphere at 37 °C. The core‐shell fibers were then cultured for 10 days.

To prepare the histogram of cells per bead, MCF‐7 cells were labeled with Cell Tracker Green (Invitrogen, USA), and cell suspensions of 2.0 × 10^4^ and 2.0 × 10^5^ cells mL^−1^ were used. After fabrication, the cells in individual beads were counted under a fluorescence microscope.

## Conflict of Interest

The authors declare no conflict of interest.

## Supporting information

Supporting Information

Supplemental Movie 1

Supplemental Movie 2

## Data Availability

Research data are not shared.

## References

[1] T. Andersen , P. Auk‐Emblem , M. Dornish , Microarrays 2015, 4, 133. 27600217 10.3390/microarrays4020133 PMC4996398

[2] T. A. Mir , M. Nakamura , Tissue Eng., Part B 2017, 23, 245. 10.1089/ten.TEB.2016.0398 28103751

[3] a) W. Shang , Y. Liu , W. Wan , C. Hu , Z. Liu , C. T. Wong , T. Fukuda , Y. Shen , Biofabrication 2017, 9, 025032; 28436920 10.1088/1758-5090/aa6ed8

[4] H. Onoe , T. Okitsu , A. Itou , M. Kato‐Negishi , R. Gojo , D. Kiriya , K. Sato , S. Miura , S. Iwanaga , K. Kuribayashi‐Shigetomi , Y. T. Matsunaga , Y. Shimoyama , S. Takeuchi , Nat. Mater. 2013, 12, 584. 23542870 10.1038/nmat3606

[5] F. Ozawa , S. Nagata , H. Oda , S. G. Yabe , H. Okochi , S. Takeuchi , iScience 2021, 24, 102309. 33997668 10.1016/j.isci.2021.102309 PMC8101052

[6] M. Sugimoto , Y. Kitagawa , M. Yamada , Y. Yajima , R. Utoh , M. Seki , Lab Chip 2018, 18, 1378. 29658964 10.1039/c7lc01280b

[7] K. Ikeda , S. Nagata , T. Okitsu , S. Takeuchi , Sci. Rep. 2017, 7, 2850. 28588295 10.1038/s41598-017-03246-2 PMC5460280

[8] K. Ino , M. T. Fukuda , K. Hiramoto , N. Taira , Y. Nashimoto , H. Shiku , J. Biosci. Bioeng. 2020, 130, 539. 32758401 10.1016/j.jbiosc.2020.06.014

[9] F. Ozawa , K. Ino , Y. Takahashi , H. Shiku , T. Matsue , J. Biosci. Bioeng. 2013, 115, 459. 23219023 10.1016/j.jbiosc.2012.10.014

[10] A. C. A. Wan , I. C. Liao , E. K. F. Yim , K. W. Leong , Macromolecules 2004, 37, 7019.

[11] B. C. Tai , A. C. Wan , J. Y. Ying , Biomaterials 2010, 31, 5927. 20472284 10.1016/j.biomaterials.2010.04.003

[12] Y. Utagawa , K. Ino , T. Kumagai , K. Hiramoto , M. Takinoue , Y. Nashimoto , H. Shiku , Micromachines 2022, 13, 420. 35334714 10.3390/mi13030420 PMC8952256

[13] M. F. Leong , J. K. C. Toh , C. Du , K. Narayanan , H. F. Lu , T. C. Lim , A. C. A. Wan , J. Y. Ying , Nat. Commun. 2013, 4, 2353. 23955534 10.1038/ncomms3353

[14] a) A. C. Wan , M. F. Leong , J. K. Toh , Y. Zheng , J. Y. Ying , Adv. Healthcare Mater. 2012, 1, 101; 10.1002/adhm.201100020 23184693

[15] M. Do , B. G. Im , J. P. Park , J.‐H. Jang , H. Lee , Adv. Funct. Mater. 2017, 27, 1702017.

[16] a) F. Wang , Z. Liu , B. Wang , L. Feng , L. Liu , F. Lv , Y. Wang , S. Wang , Angew. Chem., Int. Ed. 2014, 53, 424; 10.1002/anie.201308795 24273033

[17] D. Y. Wang , J. Duan , J. W. Liu , H. Yi , Z. Z. Zhang , H. W. Song , Y. C. Li , K. X. Zhang , Adv. Healthcare Mater. 2023, 12, 2203031. 10.1002/adhm.202203031 36708144

[18] Y. Sato , M. Takinoue , JACS Au 2022, 2, 159. 35098232 10.1021/jacsau.1c00450 PMC8790810

[19] S. H. Saxton , K. R. Stevens , J. Hepatol. 2023, 78, 873. 36038394 10.1016/j.jhep.2022.06.022

[20] F. Ozawa , K. Ino , T. Arai , J. Ramón‐Azcón , Y. Takahashi , H. Shiku , T. Matsue , Lab Chip 2013, 13, 3128. 23764965 10.1039/c3lc50455g

[21] M. Yamada , R. Utoh , K. Ohashi , K. Tatsumi , M. Yamato , T. Okano , M. Seki , Biomaterials 2012, 33, 8304. 22906609 10.1016/j.biomaterials.2012.07.068

[22] a) R. Taymour , D. Kilian , T. Ahlfeld , M. Gelinsky , A. Lode , Sci. Rep. 2021, 11, 5130; 33664366 10.1038/s41598-021-84384-6 PMC7933206

[23] J. M. d. Hoyos‐Vega , H. J. Hong , G. Stybayeva , A. Revzin , APL Bioeng. 2021, 5, 041504. 34703968 10.1063/5.0058798 PMC8519630

[24] Y. Wu , A. Wenger , H. Golzar , X. S. Tang , Sci. Rep. 2020, 10, 20648. 33244046 10.1038/s41598-020-77146-3 PMC7691334

[25] G. Yang , B. Mahadik , J. Y. Choi , J. P. Fisher , Prog. Biomed. Eng. 2020, 2, 012002. 10.1088/2516-1091/ab5637 PMC8302186 34308105

[26] L. Elomaa , M. Lindner , R. Leben , R. Niesner , M. Weinhart , Biofabrication 2022, 15. 10.1088/1758-5090/ac9433 36300786

[27] J. Gole , A. Gore , A. Richards , Y. J. Chiu , H. L. Fung , D. Bushman , H. I. Chiang , J. Chun , Y. H. Lo , K. Zhang , Nat. Biotechnol. 2013, 31, 1126. 24213699 10.1038/nbt.2720 PMC3875318

