# Supplementary material for: Fabrication and Cell Culture Applications of Core‐Shell Hydrogel Fibers Composed of Chitosan/DNA Interfacial Polyelectrolyte Complexation and Calcium Alginate: Straight and Beaded Core Variations
Source: Adv Healthc Mater. 2023 Aug 3;12(31):2302011. doi: 10.1002/adhm.202302011 (PMC11468996; doi:10.1002/adhm.202302011)
Supplement: Supplementary file 1 — Supporting Information [file ADHM-12-2302011-s001.pdf]

ADVANCED HEALTHCARE MATERIALS

Supporting Information

for *Adv. Healthcare Mater.*, DOI 10.1002/adhm.202302011

Fabrication and Cell Culture Applications of Core-Shell Hydrogel Fibers Composed of Chitosan/DNA Interfacial Polyelectrolyte Complexation and Calcium Alginate: Straight and Beaded Core Variations

Yoshinobu Utagawa, Kosuke Ino, Masahiro Takinoue and Hitoshi Shiku**

Supporting Information

Fabrication and Cell Culture Applications of Core-Shell Hydrogel Fibers Composed of Chitosan/DNA Interfacial Polyelectrolyte Complexation and Calcium Alginate: Straight and Beaded Core Variations

Yoshinobu Utagawa [1], Kosuke Ino [1,*], Masahiro Takinoue [2], and Hitoshi Shiku [1,3,**]

[1] Graduate School of Engineering, Tohoku University, Sendai 980-8579, Japan

[2] Department of Computer Science, Tokyo Institute of Technology, Yokohama 226-8502, Japan

[3] Graduate School of Environmental Studies, Tohoku University, Sendai 980-8579, Japan

Contents

-Influence of the distance between droplets on fiber formation

-Effect of Ca^{2+} and pH on cell viability.

-Effect of cell concentration in droplets on the fiber length

-Cell concentration for the hydrogel shell

-Figure S1

-Figure S2

-Figure S3

-Movie S1

-Movie S2

Influence of the distance between droplets on fiber formation

The center-to-center distance between droplets was approximately 10 mm, in the experiments included in the main text. We investigated the effect of distance between droplets on fiber formation. As shown in Figure S1A, the distance did not significantly affect the shape of a merged droplet containing an IPC film because the hydrophobic film helped smooth transferring of the droplets. After the merge, the IPC film was manually picked up to prepare an IPC fiber. There was no significant difference between fiber lengths for droplets at 10-, 20-, and 30-mm distances (Figure S1B). Although the distance did not significantly affect fiber formation in this study, several parameters affect this process. For example, a previous study reported that the hydrophobicity of the surface where droplets are set affects fiber diameter during the stage of picking fibers[1]. For precise control of droplet formations, these parameters should be considered.

Effect of Ca^{2+} and pH on cell viability

A previous study reported that a 5-min incubation with 500 mM Ca^{2+} affected cell viability, reducing the cell number to approximately 50% of the control[2]. In contrast, in the present study, the cells in a PBS droplet containing DNA contacted a chitosan droplet (pH 5.6) containing Ca^{2+}; an IPC fiber containing the cells and Ca^{2+} was formed within 30 s, and the fiber was immediately immersed in PBS (pH 7.4) with Na-alginate, resulting in the rapid removal of Ca^{2+} from the core due to diffusion and neutralization in the core. Thus, the length of time the cells spent in 500 mM Ca^{2+} and pH 5.6 was very short. To assess the impact of this protocol, we measured cell viability of HUVECs after the treatment. Because DNA/chitosan fibers were stained with propidium iodide (PI), a double staining kit using Calcein AM and PI could not be used. Also, it was difficult to centrifuge cells for removing the solution within 30 s due to the high viscosity of the solution containing chitosan. Therefore, we adhered HUVECs to a culture plate for cell viability assays. For a simple discussion, 0.5% (w/v) chitosan solution in acetate solution (pH 5.6) containing 0, 50, 100, 200, and 500 mM Ca^{2+} was used. PBS was used as control. HUVECs (5×10^3 cells/well) were seeded on a 96 well culture plates. After the cell adhesion, the cells were treated with the solution for 30 s. After the period, the solution was immediately removed, and the cells were washed with PBS three times. The cells were then cultured in a culture medium for 1 day. The cell viability was measured after 0 and 1 days using the WST-8 assay (CCK-8, Dojindo, Japan). Briefly, absorbance, showing the cell concentration, was measured using a microplate reader (SpectraMAX iD5; Molecular Devices, USA). As shown in Figure S2, there was no significant toxicity of the chitosan solution containing 100,

200, and 500 mM Ca^{2+} concentration on the cell viability on day 0, and the cell proliferation with the treatment was similar to that of the control after 1 day. The results also showed that the chitosan solution without Ca^{2+} affected the cell viability. The result indicates that the treated cells detached from the culture plate due to the low osmotic pressure of the chitosan solution. In contrast, the addition of Ca^{2+} improved the osmotic pressure during the 30 s treatment, resulting the improvement of the cell viability. In the present study, we selected 500 mM Ca^{2+} for our experiments because it provided good fiber stability without significant cell toxicity.

Effect of cell concentration in dropletson fiber length

Because only a small number of cells are incorporated into IPC fibers, high cell concentrations are desired for cell culture in the core. Therefore, we investigated the influence of cell concentration in droplets on fiber formation (Figure S3). Fiber length increased with increased cell concentration (from 0 to 1×10^6 cells/mL). The viscosity of the droplet increased with cell concentration; thus, fiber length may increase as a result of high viscosity. However, fiber length decreased when the cell concentration was further increased to 5×10^6 cells/mL. The interaction of the negative charges on the cell membrane may become relatively large at that cell concentration, leading to shorter fibers. Because the difficulty of preparation of fibers is predicted when the concentration was over 5×10^6 cells/mL, we selected the 5×10^6 cells/mL concentration for the core.

Influence of cell concentration on the hydrogel shell

In this study, we selected 500 mM Ca^{2+} for core-shell fabrication because of the good stability of the fibers; however, this meant that the shell thickness was approximately 660 µm. If the cells are densified in the shell, a shell thickness of no more than 200 µm is suitable because of the limit in oxygen and nutrient diffusion. To avoid this problem, we selected 5.0×10^5 cells/mL to scatter the cells in the shell as shown in Figure 4.

References

[1] K. Lee, M. Do, Y. C. Seo, H. Lee, *Adv. Mater. Interfaces* **2018**, *5*, 1800585.
[2] N. Cao, X. B. Chen, D. J. Schreyer, *ISRN Chem. Eng.* **2012**, 516461.

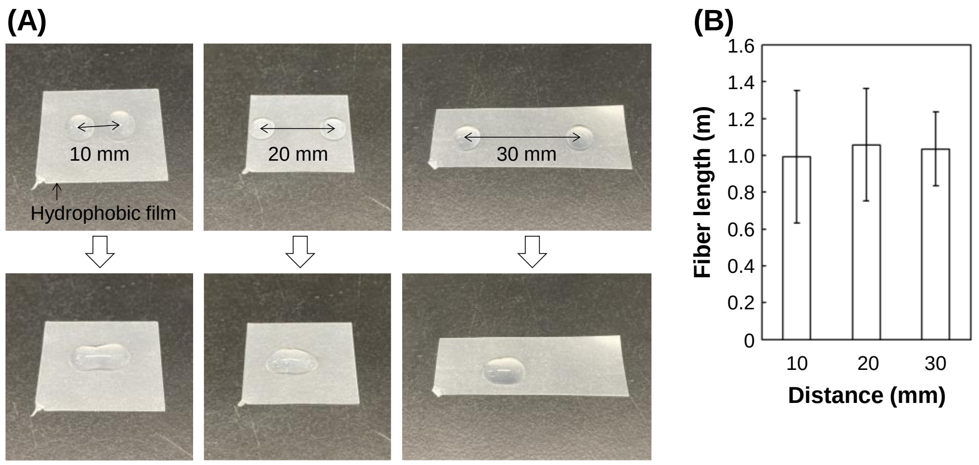

Figure S1. Influence of the distance between droplets on fiber formation. (A) Images of droplets before and after the merge. The droplets were set on the hydrophobic film, and the distance was set to be 10, 20, or 30 mm. (B) Fiber lengths when the distance was changed. The error bars represent the standard deviations. $n = 4$.

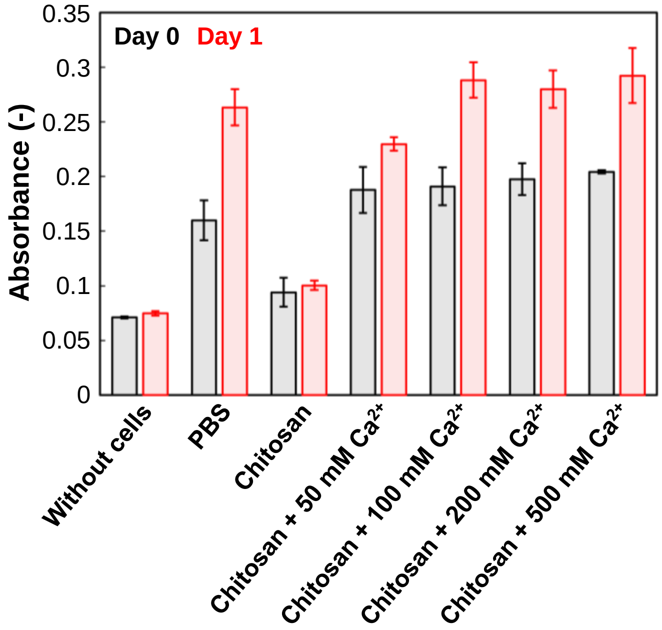

Figure S2. Effect of Ca^{2+} and pH on cell viability. Chitosan solution: pH 5.6.

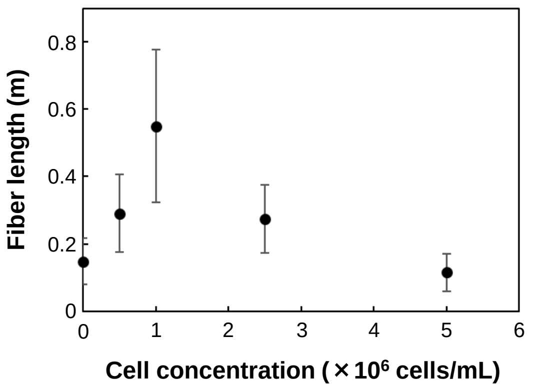

Figure S3. Influence of cell concentration in DNA droplets on fiber length. A cell suspension (0-5.0×10^6 cells/mL) of MCF-7 cells in a 0.25% (w/v) DNA solution was used. The error bars represent the standard deviations. n = 3-6.

Movie S1. Video of chitosan/DNA IPC hydrogel fibers. Chitosan:0.5% (w/v). DNA: 1.0% (w/v). Ca^{2+} was not added.

Movie S2. Video of chitosan/DNA IPC hydrogel fibers. Chitosan:0.5% (w/v). DNA: 1.0% (w/v). Subsequently, Ca^{2+} was added to the DNA droplets.
